# Magnetic nanostructuring and overcoming Brown's paradox to realize extraordinary high-temperature energy products

**DOI:** 10.1038/srep06265

**Published:** 2014-09-02

**Authors:** Balamurugan Balasubramanian, Pinaki Mukherjee, Ralph Skomski, Priyanka Manchanda, Bhaskar Das, David J. Sellmyer

**Affiliations:** 1 Nebraska Center for Materials and Nanoscience and Department of Physics and Astronomy, University of Nebraska, Lincoln, NE-68588 (USA)

## Abstract

Nanoscience has been one of the outstanding driving forces in technology recently, arguably more so in magnetism than in any other branch of science and technology. Due to nanoscale bit size, a single computer hard disk is now able to store the text of 3,000,000 average-size books, and today's high-performance permanent magnets—found in hybrid cars, wind turbines, and disk drives—are nanostructured to a large degree. The nanostructures ideally are designed from Co- and Fe-rich building blocks without critical rare-earth elements, and often are required to exhibit high coercivity and magnetization at elevated temperatures of typically up to 180 °C for many important permanent-magnet applications. Here we achieve this goal in exchange-coupled hard-soft composite films by effective nanostructuring of high-anisotropy HfCo_7_ nanoparticles with a high-magnetization Fe_65_Co_35_ phase. An analysis based on a model structure shows that the soft-phase addition improves the performance of the hard-magnetic material by mitigating Brown's paradox in magnetism, a substantial reduction of coercivity from the anisotropy field. The nanostructures exhibit a high room-temperature energy product of about 20.3 MGOe (161.5 kJ/m^3^), which is a record for a rare earth- or Pt-free magnetic material and retain values as high as 17.1 MGOe (136.1 kJ/m^3^) at 180°C.

Magnetic nanostructuring based on nanoparticle building blocks can be used to improve the performance for a variety of applications^1^,^2^,^3^,^4^,^5^. Arguably, the most important and most nontrivial magnetic property is coercivity *H*_c_. Coercivity tends to increase roughly linearly with the first magnetic anisotropy constant *K*_1_, but anisotropy is not the only consideration. Deducing *H*_c_ from *K*_1_ alone, by equating it with the anisotropy field *H*_A_ = 2*K*_1_/*M*_s_, where *M*_s_ is the saturation magnetization, tends to grossly overestimate *H*_c_, often by more than one order of magnitude^6^,^7^. This unfortunate circumstance, known as Brown's paradox, and explained by defects or nanoscale imperfections, reduces *H*_c_ to about α*H*_A_, where the “Kronmüller factor” α describes nanostructural details^8^,^9^. As-cast materials often exhibit α ≤ 0.01, and sophisticated processing methods are necessary to reach α = 0.3^10^,^11^. As in the case of bulk alloys, α « 1 means that nucleation centers or “magnetically soft spots” ruin *H*_c_. The strength of the effect is governed by the ratio *R*/δ_B_, where *R* is the radius of the harmful soft region and 
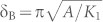
 is the Bloch-wall width of the hard phase^12^,^13^. The exchange stiffness *A* is in the range of about 1 μerg/cm (10 pJ/m) for a broad range of technical materials, whereas *K*_1_ is of the order of 50 Mergs/cm^3^ (5 MJ/m^3^) for typical hard-magnetic alloys, corresponding to a small δ_B_ ≈ 4.4 nm. The parameter α decreases with increasing ratio *R*/δ_B_, so that soft regions larger than 10 nm are usually very harmful to *H*_c_.

In this article, we develop a different way to improve *H*_c_, exploiting the fact that α increases with decreasing anisotropy constant *K*_1_. In other words, the smaller *K*_1_ leads to a larger δ_B_, and subsequently results in smaller ratio *R*/δ_B_. This approach is not usually considered, because the gain in α is overcompensated by the reduction of *H*_A_. However, top-end permanent-magnet materials such as Nd_2_Fe_14_B and SmCo_5_ contain critical rare-earth elements with raw-materials supply bottlenecks, and many alternative materials exhibit somewhat reduced anisotropy constants of the order of 10 Mergs/cm^3^ or contain expensive Pt^14^,^15^,^16^,^17^. Subject to these materials constraints, our approach focuses on Co alloys with heavy transition metals and offers the benefit of effective nanostructuring on a coarser scale of 2δ_B_ ≈ 20 nm. We achieve this nanostructuring by embedding HfCo_7_ nanoparticles in a soft Fe_65_Co_35_ phase, where the high-magnetization Fe_65_Co_35_ phase improves the net magnetization and the overall permanent-magnet performance (energy product) of the films. The fabrication of the nanostructures requires the realization of high magnetocrystalline anisotropy, easy-axis alignment, and a suitable nanostructure with high *H*_c_ and high *M*_s_. The magnetic anisotropy is often reduced in nanoparticles due to disorder and surface effects, and this leads to thermally activated magnetization reversal at high temperatures, which subsequently destroys the permanent-magnet properties^18^,^19^,^20^. By contrast, thermally activated reversal of individual nanoparticles is unimportant in the present context, because the magnetic nanoparticles are exchange-coupled in both bulk magnets and thin films.

## Results

The fabrication of our nanostructures involves two main aspects: the creation of ensembles of easy-axis aligned hard-magnetic nanoparticles and the formation of a soft-magnetic matrix. The single-step cluster-deposition process used for the sample fabrication is described elsewhere^21^,^22^, and also schematically shown as Fig. S1 in Supplementary Information. First, randomly-oriented Hf-Co nanoparticles having stoichiometry close to HfCo_7_ are produced in a water-cooled gas-aggregation chamber by sputtering a Co-Hf composite target. The high-anisotropy crystal structure in Hf-Co nanoparticles is achieved during the gas-aggregation process without a subsequent high-temperature anneal, often required in the case of wet-chemical and conventional physical vapor-deposition methods^14^,^17^,^18^,^19^,^23^. Thus, in the case of the cluster-deposition method, sintering associated with the annealing process is avoided, and thus the size-distribution and easy-axis alignment can be controlled precisely in the nanocluster-assembled nanostructures. In particular, the accomplishment of easy-axis alignment of nanoparticles in the gas-phase using a magnetic field before combining with a soft phase is an important processing step to obtain a high remanent magnetization *M_r_*, close to *M_s_*^21^,^22^,^24^,^25^. As compared to the magnetic alignment performed in the solid phase, relatively small fields of about 0.8 to 5 kOe are sufficient for the alignment of nanoparticles in the gas phase^21^,^22^,^24^,^25^. For example, in the present study, when the nanoparticles are *in-flight* in the gas phase towards the deposition chamber, the easy axes are aligned in a magnetic field of about 5 kOe before co-depositing the nanoparticles with Fe and Co atoms on Si (001) substrate using a DC magnetron sputtering gun (see Fig. S1 in Supplementary Information).

### Nanoparticle building blocks

**Figure 1:**
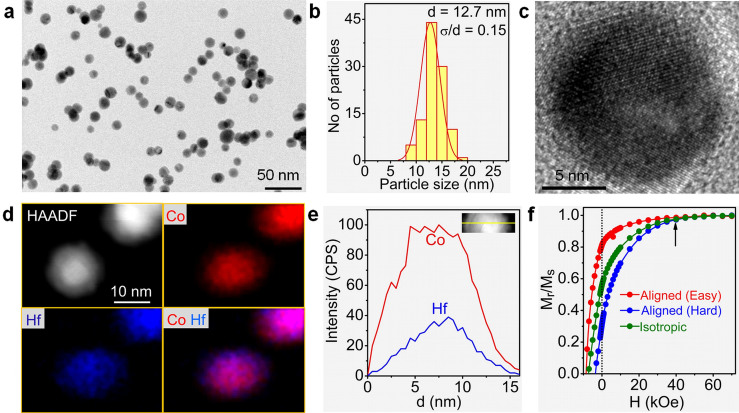
Hf-Co nanoparticles. (a), Transmission electron microscope (TEM) image. (b), The corresponding particle-size histogram (d and σ/d are the average particle size and an rms standard deviation, respectively). (c), A high-resolution TEM image of a nanoparticle. (d), A high-angle annular dark-field (HAADF) image showing the Z (atomic number) contrast and the corresponding energy-dispersive x-ray spectroscopy (EDS) color maps, showing the color distributions for Co (red), Hf (blue), and combined Co and Hf. (e), An EDS line scan showing Co and Hf distributions across a nanoparticle (shown in the inset). (f), Room-temperature hysteresis loops measured along the easy- and hard-axis directions for an aligned nanoparticle film and for an isotropic nanoparticle film.

The next step is to control the particle size, composition, crystalline ordering, and easy-axis alignment of the Hf-Co nanoparticles to achieve suitable magnetic properties, especially a high coercivity, which is a precondition to use the nanoparticles as building blocks to fabricate exchange-coupled nanocomposites. Figure 1a shows a transmission-electron-microscope (TEM) image of the Hf-Co nanoparticles that we have used to fabricate our nanocomposites. The corresponding particle-size histogram, shown in Fig. 1b, yields an average particle size of about 12.7 nm with an rms standard deviation of σ/d ≈ 0.15. The Hf-Co nanoparticles are single-crystalline with a high degree of atomic ordering, as can be seen from the high-resolution TEM image in Fig. 1c.

The composition of the nanoparticles was investigated using scanning-TEM measurements (STEM). Figure 1d shows a high-angle annular dark-field (HAADF) image of two neighboring Hf-Co nanoparticles with Z (atomic number) contrast maps and the corresponding EDS (energy-dispersive x-ray spectroscopy) color maps for Co, Hf, and the combined Co and Hf distributions. Figure 1e shows an EDS line scan measured across a single nanoparticle (shown in the inset of Fig. 1e). The STEM results reveal a uniform distribution of Hf and Co across the nanoparticles and the nanoparticles have a stoichiometry close to the HfCo_7_ phase, which crystallizes in an orthorhombic structure (see Fig. S2 in Supplementary Information for phase identification using x-ray diffraction pattern) and is also known to be magnetically anisotropic^26^.

For the magnetic measurements, the Hf-Co nanoparticles were deposited on Si (001) substrates for extended deposition times to form dense and aligned nanoparticle films. Figure 1f shows the first- and second-quadrant in-plane hysteresis loops, the magnetization *M* as a function of the applied magnetic field *H*, measured at room temperature along the easy- and hard-axis directions (see Fig. S3 in Supplementary Information for full hysteresis loops). For comparison, the room-temperature hysteresis loop of an isotropic (randomly oriented) nanoparticle film is also shown in Fig. 1f. The aligned nanoparticle film exhibits a comparatively high coercivity *H*_c_ = 8.65 kOe and a high remanence ratio *M*_r_/*M*_s_ = 0.82 along the easy axis with respect to those values measured along the hard axis (*H_c_* = 3.5 kOe and *M*_r_/*M*_s_ = 0.31) and for isotropic film (*H_c_* = 7.3 kOe and *M*_r_/*M*_s_ = 0.55). This result clearly indicates an effective easy-axis alignment in Hf-Co nanoparticles.

Figure 1f also shows that the magnetization curves merge only in a high field of about 40 kOe (indicated by an arrow). Generally a large saturation filed is required in the case of high-anisotropy nanoparticles, and also for nanoparticles that possess spin canting at interface originating from noncollinear spin structure. However, the pronounced noncollinear spin structures, as frequently observed in oxide nanoparticles and some metal nanoparticles, require competing ferromagnetic and antiferromagnetic exchange interactions^27^. In the case of strong Co-rich ferromagnetic materials, for the range of measurement field used in this study, the noncollinearity effects are generally smaller by a factor of 100–1000 as compared to the anisotropy effect^27^, and thus the observed large saturation field only suggests a fairly high HfCo_7_ magnetic anisotropy. We also have estimated *K*_1_ by fitting the magnetization curve of the isotropic Hf-Co nanoparticle film in the high-field range of about 20 to 70 kOe to the law-of-approach to saturation (Fig. S4 in Supplementary Information). This analysis yields *K*_1_ ≈ 10.6 Mergs/cm^3^, which is in the range of the reported value, *K_1_* = 14 Mergs/cm^3^, for bulk HfCo_7_ alloys^26^.

Besides having high *K_1_* and average particles size of about 12.7 nm, far less than the critical single-domain size (*D_sd_* ≈ 248 nm), the aligned HfCo_7_ nanoparticles show only *H_c_* = 8.65 kOe at 300 K. Normally the coercivity of hard magnetic nanoparticles smaller than the critical single-domain size is governed by localized nucleation at particles' surface^10^. In the meantime, the exchange-interactions between the nanoparticles also lower *H_c_*. If high-anisotropy nanoparticles are deposited on a substrate and are dilute, they cannot interact with each other and thus will behave approximately as Stoner-Wohlfarth particles with a high coercivity *H_c_* = 2*K_1_*/*M_s_*. The exchange interactions created by more dense nanoparticles generally lower *H_c_*^28^,^29^, so embedded nanoparticles in a non-magnetic matrix will maintain a relatively high *H_c_*^30^,^31^. For example, in the aligned HfCo_7_ nanoparticles, we may have some degree of exchange interactions between the particles, which decreases *H_c_* to 8.65 kOe from the Stoner-Wohlfarth value *H_c_* = 2*K_1_*/*M_s_* ≈ 24.5 kOe. However, the aim of this study is to fabricate dense hard-soft nanocomposites for obtaining strong exchange coupling to provide a high *M_s_*, while maintaining somewhat reasonable *H_c_*.

### Composite nanostructuring

**Figure 2:**
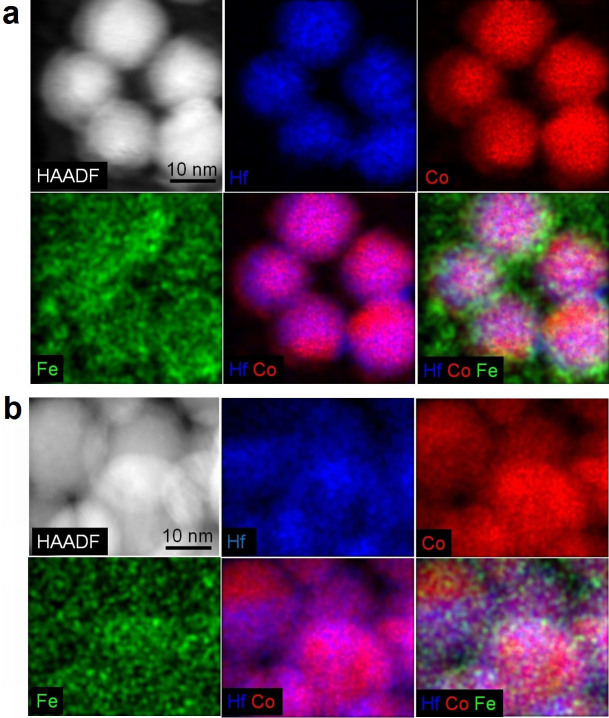
Nanostructuring. HAADF image and the corresponding EDS color maps for aligned Hf-Co:Fe-Co nanocomposite thin films having Fe-Co contents of (a), *f* = 0.22 and (b), f = 0.07. The color distributions for Hf (blue), Co (red), Fe (green), combined Hf and Co, and combined Hf, Co, and Fe are shown.

To form exchange-coupled Hf-Co:Fe-Co nanocomposite films, the aligned Hf-Co nanoparticles are co-deposited with Fe and Co atoms. Figure 2 shows the HAADF images with the Z-contrast and corresponding EDS color maps for Hf-Co:Fe-Co nanocomposite films having different volume fractions *f* of the magnetically soft Fe-Co phase, namely *f* = 0.22 (or 22 vol.%, Fig. 2a) and *f* = 0.07 (Fig. 2b). The EDS color maps, where Hf, Co, and Fe are blue, red, and green, respectively, indicate that the Fe-Co region is Fe-rich (green). The Co distribution is not visible in the matrix film due to the black background, but the individual and combined color mappings of Hf and Co shows a Co-rich region at the surface as compared to the core due to the soft Fe-Co addition (Fig. 2a). Also, x-ray diffraction analysis shows that the soft phase exhibits a body-centered cubic structure similar to that of bulk Fe_65_Co_35_ (Fig. S5 in Supplementary Information). For the samples shown in Fig. 2a and 2b, the deposition time was controlled to yield low coverage densities (thickness), to reveal the distribution of the soft phase during nanostructuring and to show that the nanoparticles are coated with and surrounded by the Fe-Co alloy.

### Magnetism of the nanocomposites

**Figure 3:**
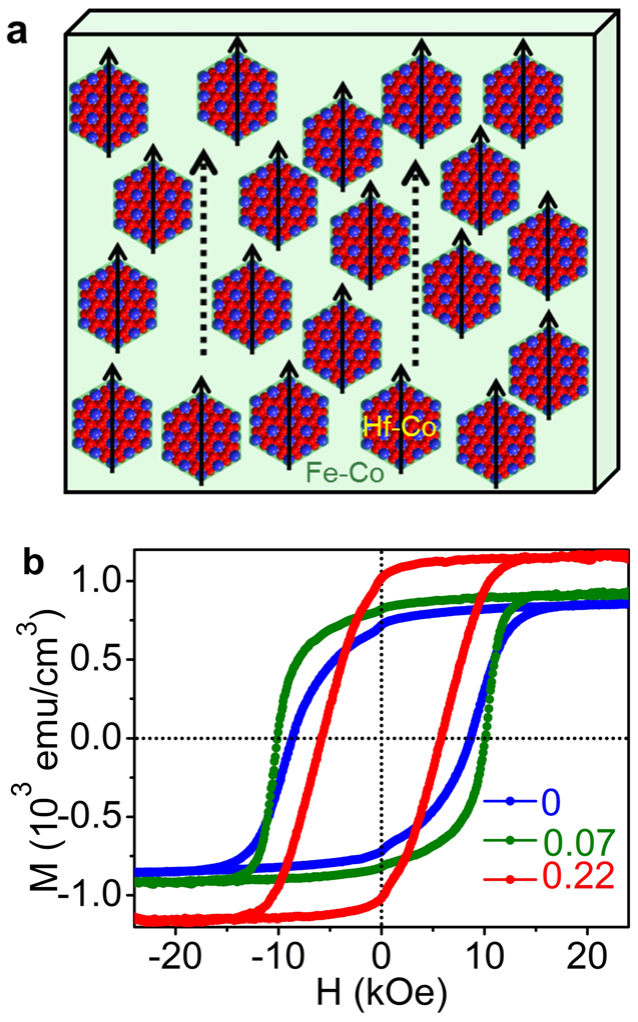
Exchange-coupled nanocomposites. (a), A schematic of the nanocomposite sample showing the dispersion of the easy-axis aligned Hf-Co nanoparticle-structures in a Fe-Co film. (b), Room-temperature hysteresis loops for Hf-Co:Fe-Co having different Fe-Co contents *f*.

The STEM results suggest that the structure of the Hf-Co:Fe-Co nanocomposite films is similar to the schematic picture of Fig. 3a. In this nanostructure, the easy-axis aligned Hf-Co nanoparticles are dispersed in an Fe-Co matrix. Except for fields near *H*_c_, the magnetization of the soft phase is parallel to the easy axis of the aligned hard nanoparticles, due to effective exchange coupling. This magnetic structure is confirmed by the room-temperature hysteresis loops measured along the easy-axis direction for the nanocomposite films. Figure 3b shows single-phase in-plane hysteresis loops for all considered volume fractions of the soft phase. A weak kink observed near *H* = 0 in the second quadrant of the hysteresis loops indicates the magnetization-reversal of the soft Fe-Co phase, originating from a weak decoupling between the soft and hard phases. The nanocomposites having *f* = 0.07 and *f* = 0.22 also exhibit high remanence ratio *M*_r_/*M*_s_ = 0.90, as compared to *M*_r_/*M*_s_ = 0.82 for bare Hf-Co nanoparticles. Like aligned HfCo_7_ nanoparticles, the aligned nanocomposite films also show high easy-axis *H_c_* and *M*_r_/*M*_s_ as compared to *H_c_* and *M*_r_/*M*_s_ measured along the hard-axis direction (Fig. S6 in Supplementary Information). Note that the room-temperature initial magnetization curves also were measured along the easy-axis direction for Hf-Co and Hf-Co:Fe-Co nanocomposite films and the results reveal a nucleation-type coercivity mechanism (Fig. S7 in Supplementary Information).

### Model structure

**Figure 4:**
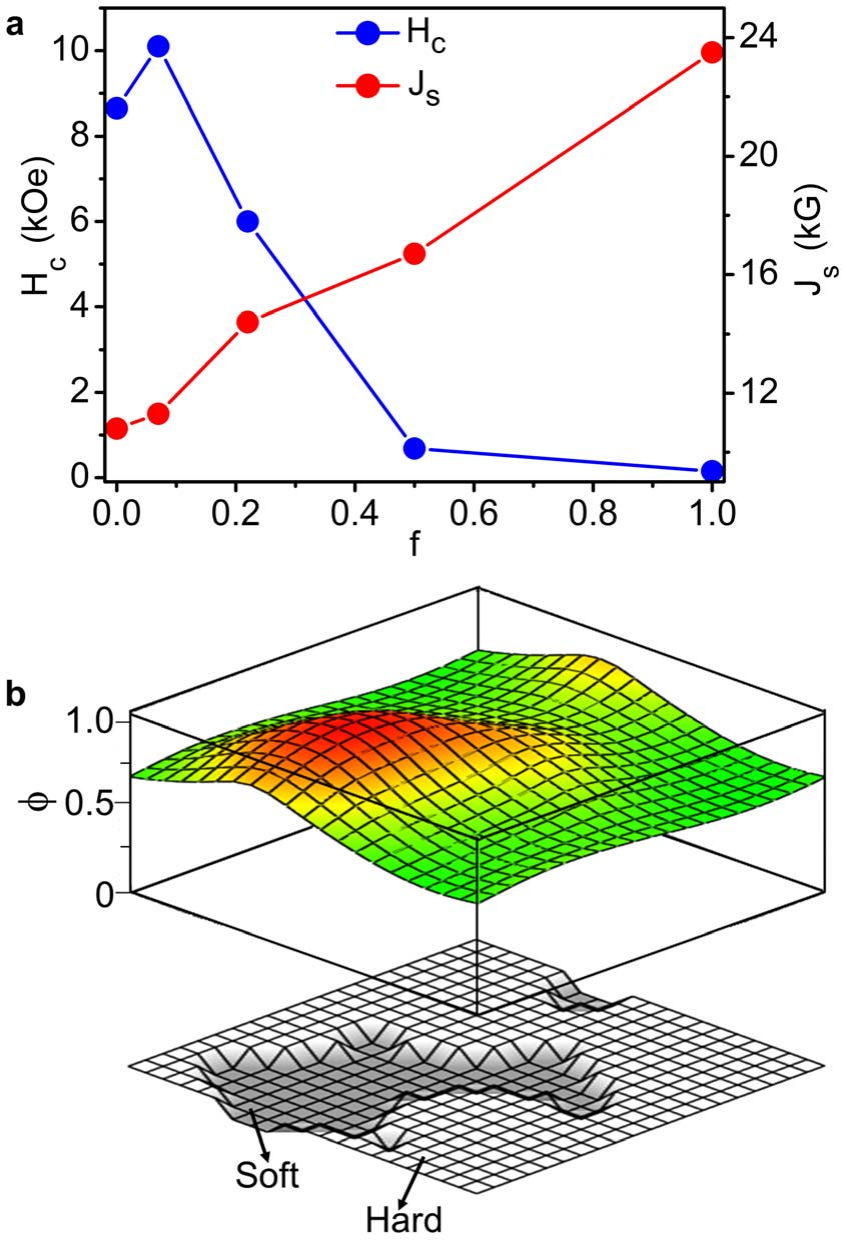
Magnetic properties. (a), Coercivity *H*_c_ and saturation magnetic polarization *J*_s_ measured at 300 K as a function of Fe-Co content *f*. (b), A thin film model structure with a total area of 10 nm × 10 nm and having a soft-phase content *f* ≈ 0.25 (bottom) and a three-dimensional visualization of the onset of magnetization reversal or nucleation mode ϕ (top).

Figure 4a shows the measured room-temperature values of the coercivity *H_c_* and of the saturation polarization *J*_s_ = 4π*M*_s_ as a function of *f*. As expected from the volume fractions of the two phases, *J*_s_ continuously increases from 10.8 to 23.5 kG as the volume fraction *f* of the soft phase increases from 0 to 1. On the other hand, *H*_c_ initially increases from 8.65 kOe to 10.1 kOe on increasing the soft phase content from 0 to 0.07, and then continuously decreases on further addition of the soft phase. The initial increase of *H*_c_ reflects the formation of the composite nanostructure and is probably caused by reduced real-structure imperfections upon surface coating and/or a comparatively better easy-axis alignment^32^.

From Fig. 4a we see that the addition of soft phase does not deteriorate the coercivity excessively, and an appreciable room-temperature value of *H*_c_ = 6 kOe was observed for Fe-Co content *f* = 0.22. In aligned hard-soft exchange-coupled nanocomposites^10^, the onset of magnetization reversal (or nucleation) is described by the nucleation mode ϕ(**r**) that defines the local magnetization angle relative to the easy axis of the hard phase. The mode obeys 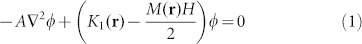
where *K*_1_(**r**) and *M*(**r**) are the local anisotropy and local magnetization, respectively. The corresponding nucleation field (coercivity) is 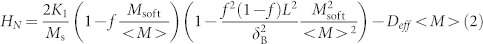
where *L*, *K*_1_, *M*_s_ are the average center-to-center distance, magnetic anisotropy constant, and saturation magnetization of the hard particles, respectively. *M*_soft_ represents the saturation magnetization of the soft phase and <*M*> = (1 − *f*) *M*_s_ + *f* *M*_soft_ is the average saturation magnetization. In equation (2), the product of the two terms in parentheses is equal to α, and the demagnetization factor *D_eff_* is ideally zero for thin films subjected to an in-plane magnetic field *H* applied parallel to the anisotropy easy axis, as in the present case. The main correction (to α = 1) comes from the ratio 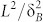. Since 
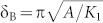
 is relatively large for HfCo_7_, about 12 nm, α is fairly large. Note that the equation (2) is limited to small-scale nanostructures (*L* smaller than about 20 nm.)

In order to understand the magnetization reversal, we have also used equation (1) to estimate the nucleation mode numerically for an ideal thin-film model structure, shown in Fig. 4b, which is similar to our nanocomposite films. Note that, by definition, the soft phase is already included in calculating the parameter α for the model structure shown in Fig. 4b, and a good example is Kronmüller's original analysis^8^, where the main effect comes from a built-in soft region. The model structure (bottom image in Fig. 4b) has a total area of 10 nm × 10 nm with *f* = 0.25 and creates a nucleation mode (top image of Fig. 4b) that is centered around the soft region but penetrates deep into the hard region. If the nanocomposite films were macroscopic rather than nanoscale, then the nucleation mode would be localized almost entirely in the soft regions and lead to very low *H*_c_. However, in small-scale nanostructures, such as the present ones, the mode is largely delocalized—extending into the hard phase—and therefore benefits from the admixture of the anisotropy of the hard grains. This penetration means that the hard phase micromagnetically stabilizes the soft phase and realizes a nearly uniform nucleation mode.

The corresponding penetration depth is of the order of δ_B_^12^,^13^ and increases with decreasing *K*_1_, leading to a tradeoff between enhanced exchange length (~1/*K*_1_^1/2^) and reduced anisotropy field (~*K*_1_). From a simplistic viewpoint, the high anisotropy always wins. However, in reality, Brown's paradox states in effect that *H*_c_ is usually much smaller than *H*_A_. The paradox is explained by defects^6^,^8^,^12^ and leads to a coercivity reduction to about α*H*_A_^8^,^12^. For example, the numerical calculation yields α ≈ 0.70 for the ideal nanostructure shown in Fig. 4b, and thus the nanocomposite structure is expected to retain considerable coercivity from the hard phase. For example, in supporting this trend, our Hf-Co:FeCo nanocomposite film with *f* = 0.22 almost retains 69% of the hard-phase coercivity.

### High-temperature performance

**Figure 5:**
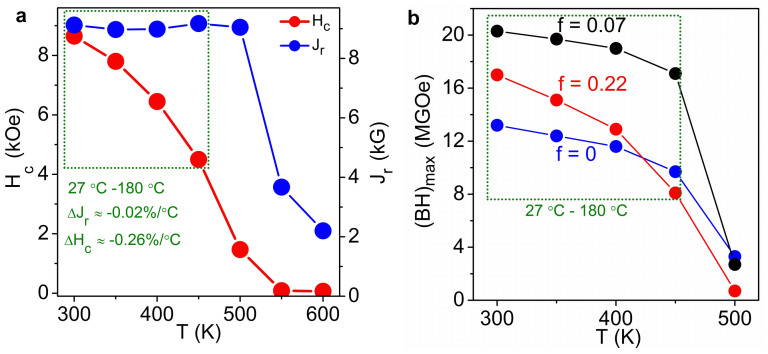
High-temperature performance. (a), Temperature-dependent coercivity *H*_c_ and remanence *J*_r_ for Hf-Co nanoparticles. Δ*H*_c_ and Δ*J*_r_ denote the temperature coefficients in the temperature range of 27°C to 180°C. (b), The measured energy products (*BH*)_max_ for nanocomposite films having different soft Fe-Co phase content *f*. (*BH*)_max_ is the maximum value from the second quadrant of the *BH* curve (*B = H + 4πM* is the magnetic flux density). The dotted green rectangles in (a) and (b) mark the typical temperature region of 27 °C to 180 °C.

Permanent magnets often are required to operate above room temperature, for example at about 180 °C in high-performance motors, and it is important that remanent magnetic polarization *J*_r_ and coercivity *H*_c_ remain high at the operation temperature. The same applies to the energy product, a key figure of merit that describes the magnet's ability to store magnetostatic energy in free space^12^. Of crucial importance in this regard are the reversible temperature coefficients Δ*J*_r_ = d*J*_r_/d*T* and Δ*H*_c_ = d*H*_c_/d*T* of remanence and coercivity^33^. For example, Fig. 5a shows the measured values of *H_c_* and *J_r_* for Hf-Co nanoparticles as a function of temperature. The Hf-Co nanoparticles exhibit appreciable coercivities (from 8.65 to 4.5 kOe) and remanences (from 9.2 to 9.0 kG) for the temperature range of 27 °C to 180 °C. Note that *J_r_* shows a slight increase to 9.2 kG at 450 K as compared to *J_r_* = 9.1 kG (at 300 K) and *J_r_* = 9.0 kG (at 400 K) and this variation is within the experimental error in magnetization. Figure 5a yields very low average temperature coefficients Δ*J*_r_ ≈ −0.02%/°C and Δ*H*_c_ ≈ −0.26%/°C for HfCo_7_ nanoparticles, which are far superior to the values obtained for the leading high-performance permanent-magnet material Nd_2_Fe_14_B (Δ*J*_r_ = −0.10%/°C and Δ*H*_c_ = −0.40%/°C)^33^,^34^,^35^. This result can be attributed to the presence of about 87.5 at% of high Curie temperature Co in HfCo_7_ nanoparticles. Similar trends are found in the nanocomposites, such as high coercivities of 10.1 and 4.2 kOe (*f* = 0.07) and of 6.0 and 1.9 kOe (*f* = 0.22) in the temperature range of 27°C–180°C. Note that *K*_1_ decreases with increasing temperature, which further enhances δ_B_ and makes the nanostructuring more effective.

### Discussion

The low temperature coefficients translate into a favorable temperature dependence of the nominal energy product corresponding to the mass of the magnetic materials in the films (Fig. 5b). For example, the nanocomposite having *f* = 0.07 exhibits energy products of 20.3 MGOe (161.5 kJ/m^3^) at 27 °C and 17.1 MGOe (136.1 kJ/m^3^) at 180 °C. 20.3 MGOe at room temperature is the record energy product for a critical rare earth- or expensive Pt-free material^16^,^21^,^36^,^37^. In addition, the energy product, 17.1 MGOe, obtained at 180 °C is the highest reported value for non-rare-earth or Pt-free materials. FePt-based alloys have shown a high energy product at room temperature (~54MGOe)^38^. However, to the best of our knowledge, high-temperature energy products have not been reported for FePt. The excellent high-temperature performance in our Co-rich nanostructures is achieved in spite of the fact that the anisotropy of the hard HfCo_7_ nanoparticles is relatively modest, by overcoming Brown's paradox through nanostructuring. Also, for the first time, the potential of nanoparticles for fabricating high-temperature permanent-magnet materials is demonstrated. Naturally, for bulk magnetic applications, scale-up methods to create such nanostructures remain a significant challenge, but our approach provides useful insights for developing future rare-earth-free permanent magnets.

## Methods

The aligned Hf-Co nanoparticles and the Hf-Co:Fe-Co nanocomposites were produced using a cluster-deposition method described elsewhere (also see the supplementary information S1)^22^,^21^. Some of the nanoparticles were deposited on carbon-coated copper grids with low coverage densities to measure the average particle size and size distribution using a FEI Tecnai Osiris (Scanning) transmission electron microscope. For the SQUID (superconducting quantum interference device) and PPMS (physical property measurement system) measurements, thin films of dense-packed nanoparticles and nanocomposites were fabricated on single-crystalline Si (001) substrates and the error in evaluating the coercivity and magnetization from the hysteresis loops is within 2%. The mass (or nominal thickness) of the films was measured using a quartz-crystal thickness monitor. The thickness of the films reported in this study was always in the range of 110 - 280 nm. The nanoparticle/nanocomposite thin films were capped with a thin SiO_2_ cap layer immediately after deposition by using a radio-frequency magnetron sputtering gun located in the deposition chamber. X-ray diffraction measurement of Hf-Co nanoparticles deposited on Si (001) substrate was carried out using a Rigaku D/Max-B diffractometer with a Co K_α_ wavelength of about 1.7889 Å.

## Author Contributions

B.B., D.J.S. and R.S. developed the concept, and B.B. and D.J.S. designed the experiments. B.B. carried out the preparation and characterization of the nanocomposites samples. P.M. (Pinaki Mukherjee) carried out the TEM measurements. R.S. and P.M. (Priyanka Manchanda) performed the model calculations. B.B., R.S. and D.J.S. analyzed the results and wrote the manuscript. B.D. contributed to the preparation of the bulk Fe_65_Co_35_ alloys. All authors critically read and commented on the manuscript.

## Supplementary Material

Supplementary Information Figure S1-S7
