# Supplementary material for: Magnetic nanostructuring and overcoming Brown's paradox to realize extraordinary high-temperature energy products
Source: Sci Rep. 2014 Sep 2;4:6265. doi: 10.1038/srep06265 (PMC4151151; doi:10.1038/srep06265)
Supplement: Supplementary Information — Figure S1-S7 [file srep06265-s1.pdf]

Supplementary Information

Magnetic nanostructuring and overcoming Brown's paradox to realize extraordinary high-temperature energy products

Balamurugan Balasubramanian, Pinaki Mukherjee, Ralph Skomski, Priyanka Manchanda, Bhaskar Das, and David J. Sellmyer

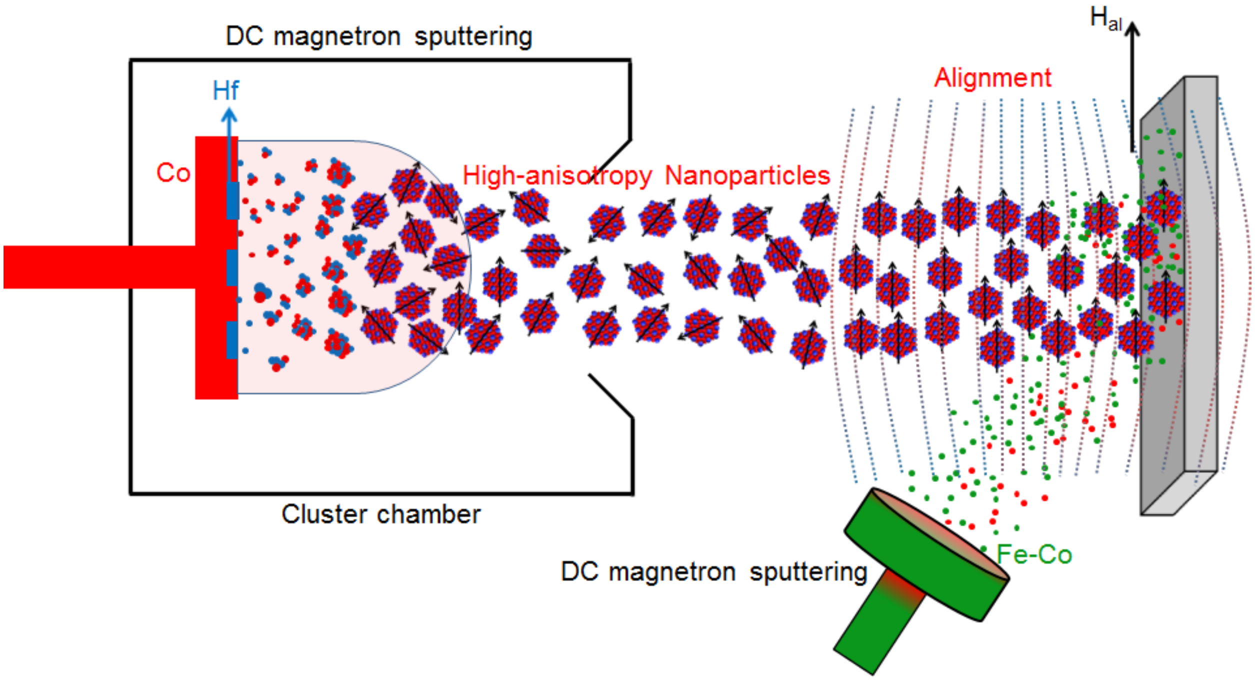

Figure S1 | Cluster-deposition method. The process for fabricating the exchange-coupled nanocomposites includes the production of high-anisotropy Hf-Co nanoparticles, the alignment of their easy axes using a magnetic field $H_{al} \approx 5$ kOe, and the Co-deposition of Fe and Co atoms to form a high-magnetization Fe-Co phase.

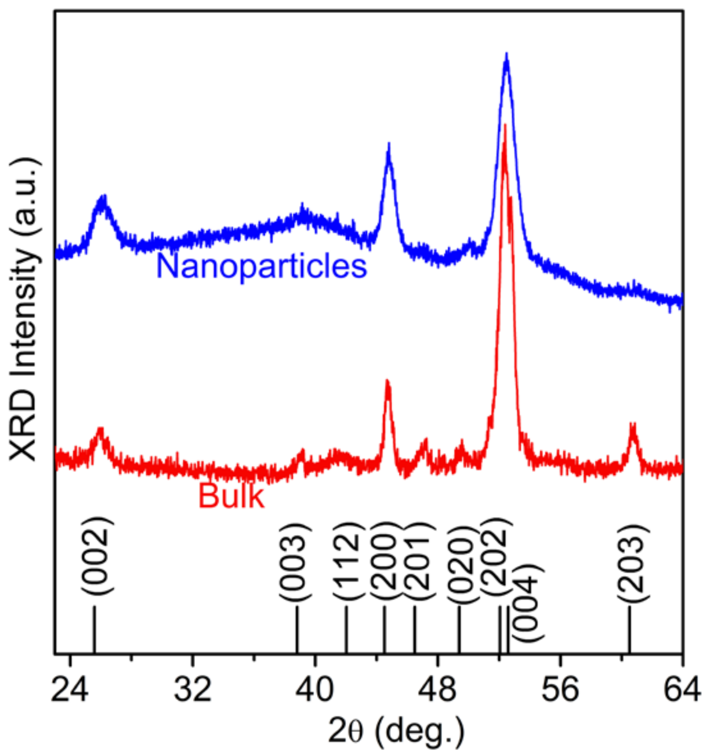

Figure S2 | Structure of $HfCo_7$. X-ray diffraction (XRD) pattern of the nanoparticles is compared with that of bulk alloy having orthorhombic structure. XRD pattern of the bulk alloy is adapted from ref 1 and corrected for Co K_α radiation having a wavelength of about 1.7889 Å. The XRD peak positions determined using TOPAS (Total Pattern Analysis Solution, Bruker AXS) for an orthorhombic structure having lattice parameters of about a = 4.719 Å, b = 4.278 Å, and c = 8.070 Å is shown as vertical lines.

Reference

1. Das, B. et al. $HfCo_7$-based rare-earth-free permanent-magnet alloys. *IEEE Trans. Magnet.* **49**, 3330-3333 (2013).

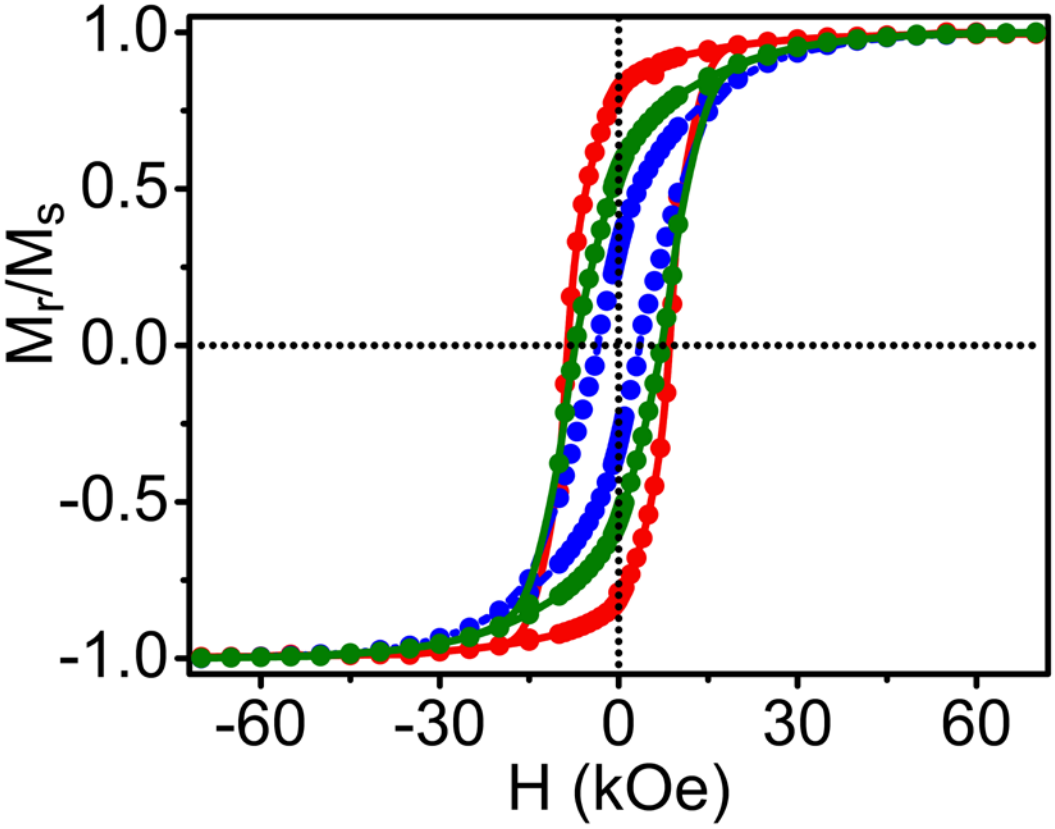

Figure S3 | $HfCo_7$ nanoparticles. Room-temperature in-plane hysteresis loops measured along the easy- (red) and hard-axis (blue) directions for an aligned nanoparticle film and for an isotropic nanoparticle film (green).

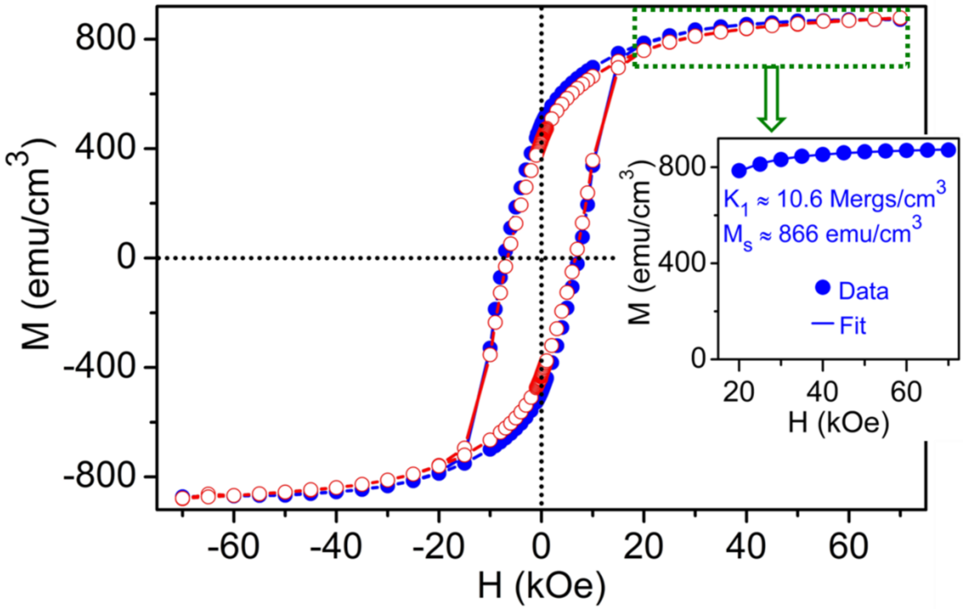

Figure S4 | Analysis of approach to saturation. Room-temperature hysteresis loops of isotropic (unaligned) Hf-Co nanoparticles measured along the in-plane (blue) and out-of-plane (red) directions. To estimate the magnetic anisotropy constant K_1 by following the law-of-approach to saturation,[1,2] the magnetization near saturation (M_s) in the field range of 20 – 70 kOe for the in-plane magnetization curve is fitted, as shown in the inset, using the law-of-approach to saturation, $M = M_s\,(1 - c/H^2) + \chi H$. Here χ is the high-field susceptibility and the constant c depends on the anisotropy constant K_1 as given by $c = \frac{4}{15}\frac{K_1^{\,2}}{M_s^{\,2}}$. This analysis yield $K_1 \approx 10.6$ Mergs/cm^3.

References

1. Hadjipanayis, G., Sellmyer, D.J., & Brandt, B. Rare-earth-rich metallic glasses. I. magnetic hysteresis. Phys. Rev. B **23**, 3349-3354 (1981).
2. Kneller, E. *Ferromagnetisms* (Springer, Berlin, 1962).

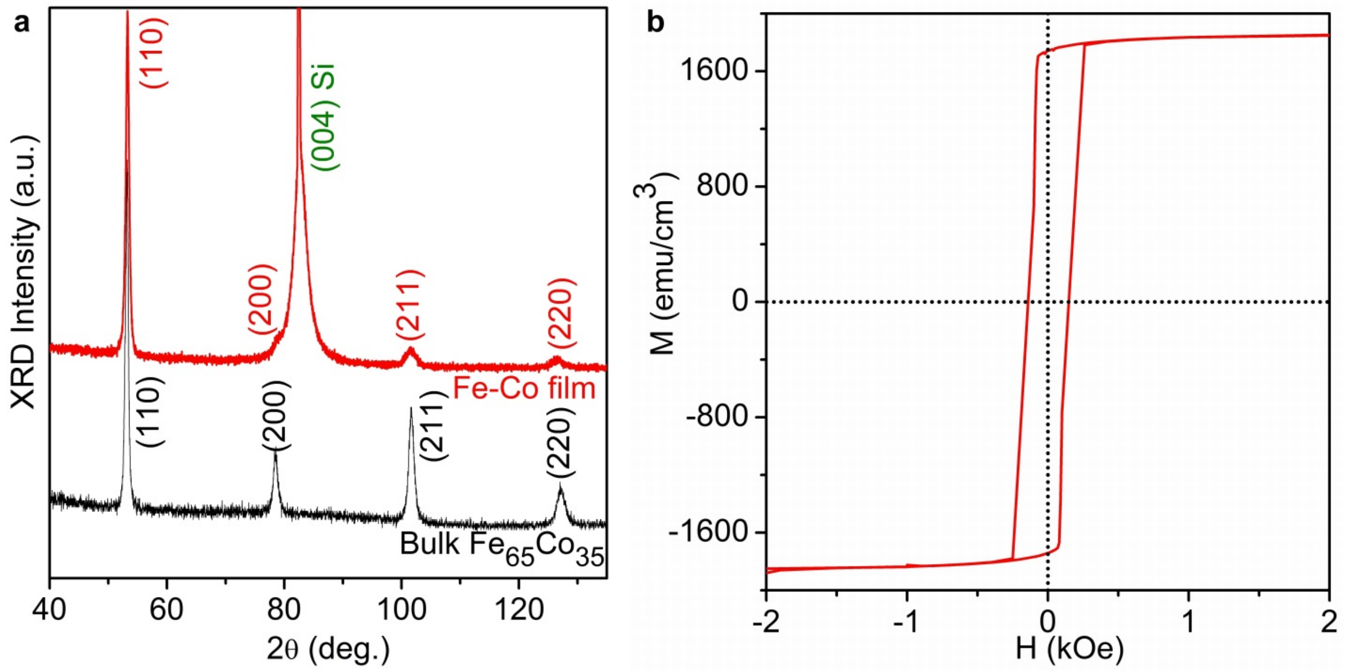

Figure S5 | Soft Fe-Co phase. a, X-ray diffraction pattern of a Fe-Co matrix film is compared with that of a bulk $Fe_{65}Co_{35}$ alloy having a body centered cubic structure. **b,** Room-temperature hysteresis loop of the Fe-Co matrix film. The saturation magnetization M_s measured from the loop is about 1873 emu/cm^3. For these measurements, Fe-Co matrix film is deposited on Si (001) substrate in the absence of the cluster beam.

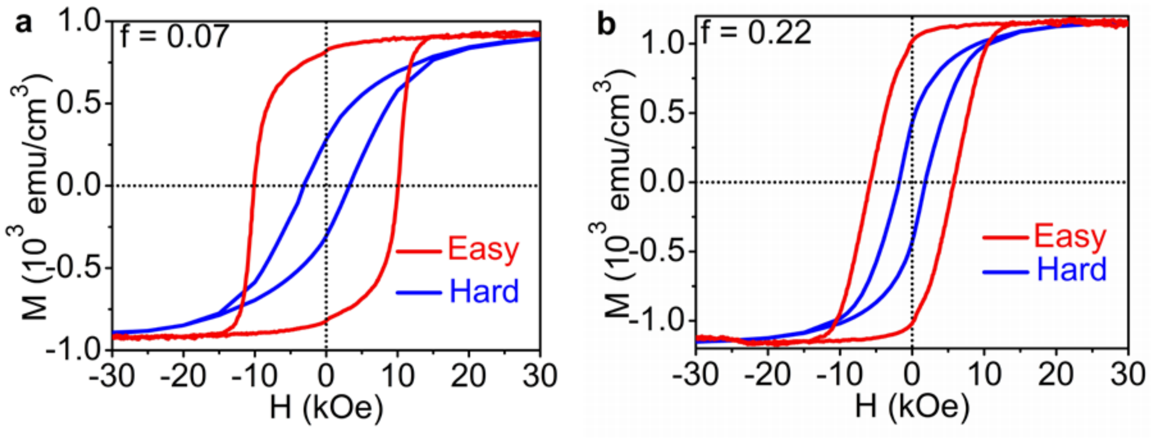

Figure S6 | Hf-Co:Fe-Co nanocomposite films. Room-temperature in-plane hysteresis loops measured along the easy- and hard-axis directions for different Fe-Co contents: **a,** f = 0.07. **b,** f = 0.22.

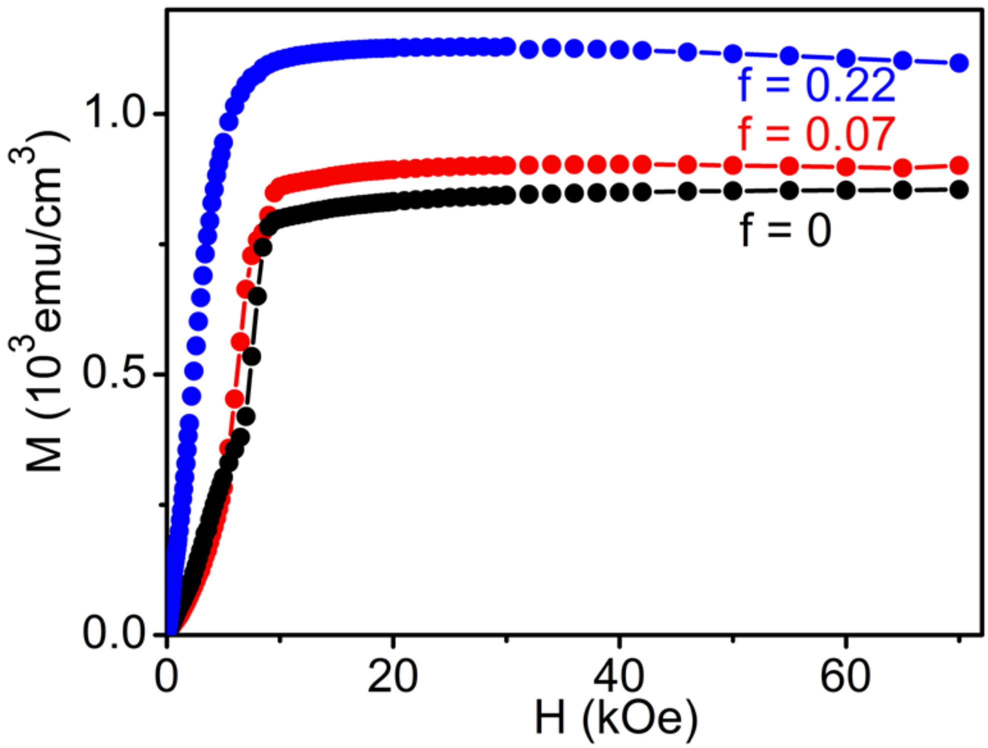

Figure S7 | Hf-Co:Fe-Co nanocomposites. Initial room-temperature magnetization curves measured along the easy axis for different Fe-Co contents *f*. Note that the as-deposited aligned nanocomposite films are already in remanent state, and thus they are demagnetized to attain virgin state before measuring the initial magnetization curves.
